# The transformative impact of AI-enabled AlphaFold 3: evolution, current status, and future prospects in structural biology

**DOI:** 10.3389/frai.2026.1739303

**Published:** 2026-04-08

**Authors:** Chiranjib Chakraborty, Manojit Bhattacharya, Sang-Soo Lee

**Affiliations:** 1 Department of Biotechnology, School of Life Science and Biotechnology, Adamas University, Kolkata, India; 2 Centre of Excellence for Advanced Medical Bioinformatics and Artificial Intelligence, Adamas University, Kolkata, India; 3 Department of Zoology, Fakir Mohan University, Balasore, India; 4 Institute for Skeletal Aging & Orthopedic Surgery, Hallym University-Chuncheon Sacred Heart Hospital, Chuncheon-si, Republic of Korea

**Keywords:** AlphaFold, CASP, protein structure prediction, revolution in application, structural biology

## Abstract

The AlphaFold (AF) initiative profoundly impacted structural biology, evidenced by its 2024 Nobel Prize. AlphaFold progressed from AF1 to AF2, which achieved near-experimental accuracy in single-chain protein folding, and then to AF3, expanding predictions to protein-ligand, protein-nucleic acid, and protein–protein complexes. This evolution led to the widespread adoption of AF tools, expanded structural coverage, and greater accessibility through the AlphaFold Database (AFDB), accelerating translational research, especially in structure-based drug discovery (SBDD) and the study of complex macromolecular assemblies. AF1 uses deep neural networks (DNNs), AF2 employs the Evoformer to model evolutionarily related sequences, and AF3 applies the Pairformer for pairwise amino acid interactions. The main differences between AF versions are architectural. Remaining challenges include predicting protein dynamics and multiple conformational states. This review first outlines AlphaFold’s architectural evolution, then explores the post-AlphaFold landscape and its global impact, discusses translational research applications, and addresses limitations and future directions. Despite challenges, AlphaFold is poised to further advance structural biology, particularly in biotechnology and medicine.

## 1 Introduction

**Figure 1:**
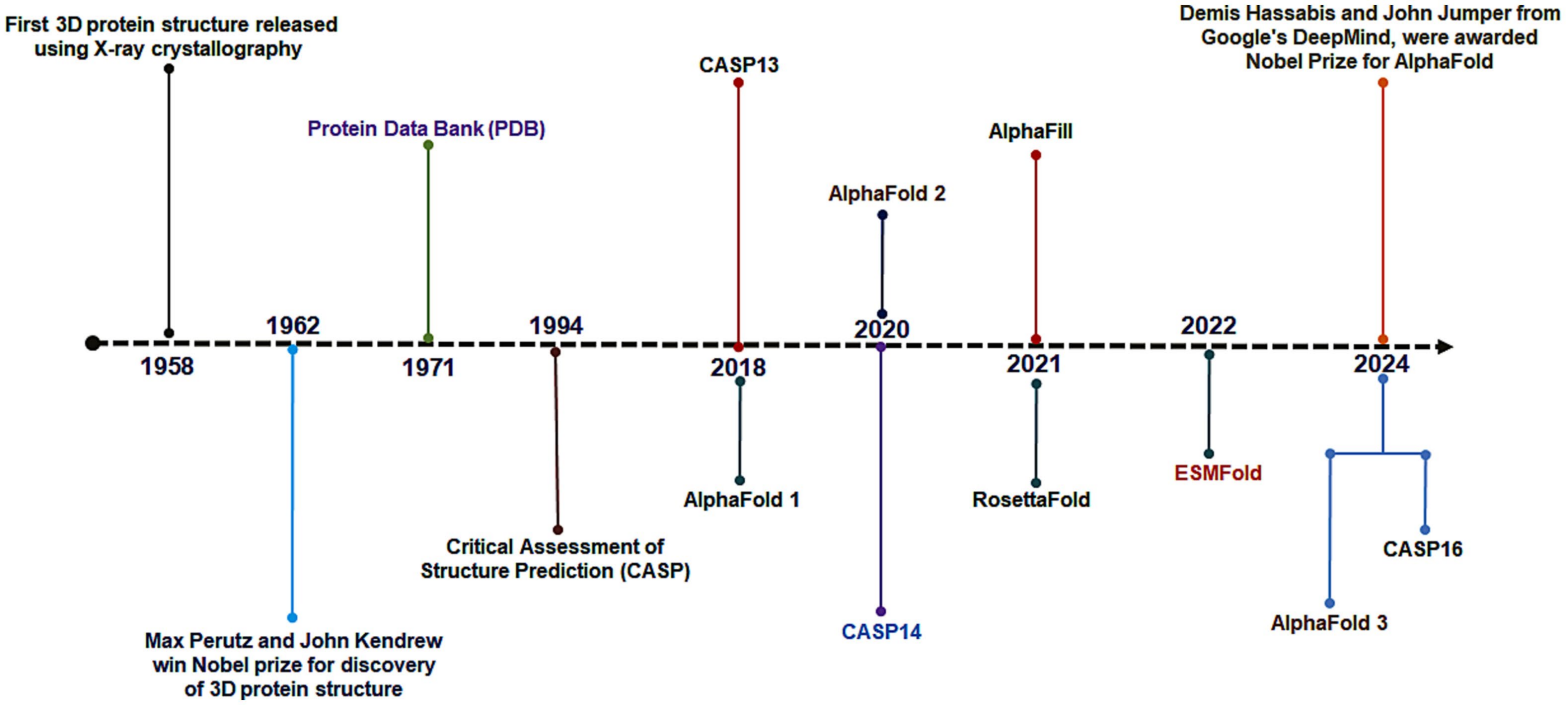
The figure illustrates the timeline of structural biology development, from the inception of the Protein Data Bank (PDB) to the emergence of AlphaFold.

One of the major challenges in structural biology is determining the three-dimensional conformation of proteins, which defines their function. The field advanced in the 1950s with techniques like X-ray crystallography (Shi, 2014), culminating in John Kendrew’s determination of the first protein structure (myoglobin) in 1958. In 1962, Max Perutz and John Kendrew received the Nobel Prize for their pioneering work (Jaskolski et al., 2014). Increasing structural data led to the establishment of the Protein Data Bank (PDB) at Brookhaven National Laboratory in 1971, which became the central global archive for protein structures (Figure 1). The open-access model of the PDB fostered broad research usage and critical data sharing (Kurisu, 2022; Berman and Burley, 2025). Long-term support by the NSF, NIH, and DOE, with NIH as the main contributor, ensured PDB’s impact on subsequent advances, including AlphaFold (Burley et al., 2022; Kurisu, 2022). Despite the PDB’s growth, the gap between known sequences and solved structures widened due to the limitations of experimental methods. This led to the launch of CASP in 1994, a biennial challenge for computational prediction of protein structure (Staples et al., 2019). Traditional methods, mainly homology modeling and ab initio methods, relied on metrics such as the Global Distance Test (GDT) to measure model accuracy (Kryshtafovych et al., 2019; Zemla, 2003; Bieri and Kiefhaber, 1999; Li et al., 2016). By 2016, these techniques reached a GDT of about 40/100 for the most difficult targets, showing major computational limitations (Anishchenko et al., 2014; Hameduh et al., 2020; Wuyun et al., 2024). A breakthrough arrived when Google DeepMind’s AlphaFold, introduced at CASP13 in 2018, used deep learning and coevolutionary information to accurately predict protein structures, ranking first in the Free Modeling category (AlQuraishi, 2019; Senior et al., 2020).

In 2020, AlphaFold 2 (AF2) by DeepMind showed excellent accuracy in the CASP14 competition. Experts stated that AF2 achieved high accuracy in predicting single-chain protein structures, with a GDT score of about 92.4%. This breakthrough has revolutionized structural biology research (Perrakis and Sixma, 2021; Callaway, 2022; Editorial, 2022). As a result, protein structure prediction has since shifted toward more complex structures. Researchers consequently focus on challenges such as predicting protein interactions, molecular binding, and conformational changes, which are actively shaping the field’s future. In this review, we first outline AlphaFold’s architectural trajectory from co-evolution to universal modelling and discuss the post-AlphaFold landscape and its global impact. We then illustrate AlphaFold’s translational research applications. Finally, we discuss limitations, next-generation challenges, and the future trajectory.

## 2 AlphaFold: from co-evolution to universal modelling

Each version of AlphaFold embodies a distinct architectural strategy, meticulously designed to address increasingly complex challenges in biomolecular prediction. We have described each version of AlphaFold, which is as follows:

### 2.1 AlphaFold 1 (CASP13): deep learning (DL) for contact prediction

AlphaFold 1 (AF1) aimed to predict which parts of a protein are physically close in the CASP13 competition. The researchers used two main approaches: first, they compared many similar protein sequences to identify regions that change together, suggesting these parts likely interact in the 3D structure. Second, they used deep learning methods to study these relationships. Then, they turned this data into maps showing which parts of the protein are close together (Hou et al., 2019). AF1 built the final 3D shapes using these advanced algorithms. This success shows how artificial intelligence can help us understand protein structures (AlQuraishi, 2019; Hou et al., 2019).

### 2.2 AlphaFold 2 (CASP14): deep learning (DL) revolution

The AF2 was significantly improved from the previous version. It utilized a DNN to generate protein structures from amino acid (AA) sequences. At the same time, it also utilizes AF3 capabilities, multiple sequence alignments (MSA), and related proteins (Pratt et al., 2025). The central part of the analysis was performed using Evoformer (Jumper et al., 2021b). It worked with the MSA data by assigning different weights to different areas, which helped manage complex, long-range connections within the structure. This mechanism, known as attention, allows the Evoformer to focus computational resources by assigning weights to different parts of the input data. Specifically, Evoformer uses axial or criss-cross attention, which applies row-wise gated self-attention within each sequence and column-wise gated self-attention across sequences in the MSA. This approach enables the model to prioritize relevant information when analyzing data. The performance of AF2 was assessed using the TM-score, which ranges from 0 to 1. Usually, a score exceeding 0.4 signifies structural similarity (Schlievert et al., 1996; Zhang and Skolnick, 2005; Skolnick et al., 2021; Pratt et al., 2025). AF2 achieved a remarkable 92.4 GDT, and it can generate models nearly as precise as those derived from experimental methods (Skolnick et al., 2021). These accurate models were promptly integrated into traditional experiments. It facilitates the determination of structures in X-ray crystallography by providing a useful starting point for molecular replacement. In addition to X-ray crystallography-based structures in PDB, NMR structures in PDB were also included, which caused a complication. These structures are essential for the rapid growth of structural data. However, it introduces complications primarily because NMR relies on a previously known, similar structure as a template (the search model) to determine an unknown structure (Abergel, 2013; Scapin, 2013).

There has been a recent protein structural revolution, driven by cryo-EM. About 40% of the new structures deposited into the PDB from 2024 to 2025 were obtained using this technique (Rubach et al., 2025; Berman and Burley, 2025; RCSB PDB, 2026). Similarly, NMR structures and cryo-EM-based structures have also been instrumental in creating or training using AF. Therefore, the combined impact of AI-driven modeling (such as AF) and experimental techniques, including cryo-EM, X-ray crystallography, and NMR. It has led to a record-breaking number of new structures in 2025–2026.

TM-score and GDT are related but distinct metrics for protein structure similarity. The TM-score ranges from 0 to 1. It is widely used for pairwise structural comparison and is less sensitive to protein length (Xu and Zhang, 2010). GDT, most often reported as GDT_TS (%), is the standard metric in CASP experiments for predicting structure accuracy (Moult et al., 2003). While TM-score often measures overall agreement, AlphaFold2’s CASP14 performance was measured using GDT_TS. AF2 achieved a median of about 92.4, indicating near-experimental accuracy (Jumper et al., 2021b). We shift from TM-score to GDT here to align with the official CASP14 evaluation, not to introduce inconsistency.

### 2.3 AlphaFold 3: universal prediction of biomolecular interactions

Google DeepMind and Isomorphic Labs released AlphaFold 3 (AF3) on May 8, 2024. It represents a substantial advancement in both its design and scope. AF3 can model the structures of complexes that include not only proteins but also small molecules, ions, DNA/RNA, and modified residues (Fang et al., 2025; Hennig, 2025; Krokidis et al., 2025). This shift toward a comprehensive model for all molecular entities underscores the idea that molecular function depends on interactions rather than solely on individual protein structures. In the architecture, AF2 uses the Evoformer module. At the same time, in the architecture, AF3 uses a simplified module called Pairformer. This modification facilitates the processing of extensive MSAs while maintaining or enhancing prediction accuracy, particularly for complex structures (Abramson et al., 2024; Krokidis et al., 2025). By implementing token-based spatial reasoning across various molecular types, the system achieves greater generality and efficiency in modelling interactions. Although tokens are not a major novelty of AF3, they were already used in AF2. The novelty of AF3 is Pairforme. It replaces the Evoformer with a “Pairformer” module. It operates exclusively on token pairs and employs an “Atom Transformer” (also known as Atom Attention Encoder) to manage interactions between atoms. The advancement AF3 has led to the discovery of novel pharmaceuticals by utilizing the unsolved structure of drug targets. Actually, at present, drug discovery is increasingly using drug targets and their structures. If the drug target’s structure is unavailable, the drug discovery process is hindered, and the researcher may use molecular modeling to build a model of the drug target. In this case, AF3 has solved several previously unsolved protein structures, which can serve as drug targets. AF presents a big promise, but it has not led to new drugs to date. Several AF-inspired projects have been developed in this direction (pre-clinical, clinical) (Table 1). Similarly, AF3 demonstrates exceptional proficiency in predicting drug interactions, including the binding of proteins to other molecules and the binding of antibodies to target proteins (Desai et al., 2024; Shi, 2024). A PoseBusters assessment is an evaluation of predicted protein-ligand binding positions. It understands the priority of binding positions in terms of physical and chemical feasibility, as well as geometric accuracy (Buttenschoen et al., 2024). In the challenging PoseBusters assessment, AF3 demonstrated a 50% improvement in accuracy over the most advanced traditional physics-based methods. Without any structural input, this was performed (Krokidis et al., 2025). PoseBusters is a Python package. It performs a series of standard quality checks. These checks use the well-established cheminformatics toolkit RDKit. The test suite confirms the chemical and geometric consistency of a ligand. It includes its stereochemistry (Buttenschoen et al., 2024). In this experiment, PoseBusters was used. Without any structural input, this was performed. The protein-ligand complex was predicted solely from the protein and ligand sequences and chemical SMILES strings. It was not predicted by docking into a known receptor structure (Buttenschoen et al., 2024; Lee et al., 2025a). It was the first example of an AI using physics-based tools to predict biomolecular structures in this domain, demonstrating its ability to predict protein-ligand binding positions and achieve geometric accuracy. The progression of AlphaFold across its three major versions is summarized in Table 2.

**Table 1:** AlphaFold-inspired projects related to pharmaceuticals and their status.

Sl. No	Project (pharmaceuticals)	Stage/status	Reference
1.	Insilico Medicine – CDK20 inhibitor	Pre-clinical	(Ren et al., 2023)
2.	Viral target screening (e.g., NSP6, SARS-CoV-2)	Pre-clinical	(Desai et al., 2024)
3.	Academic/de novo design tools (PCMol, generative methods)	Early discovery	(Bernett et al., 2024)

**Table 2:** Comparative evolution and benchmarks of AlphaFold systems.

AlphaFold version	CASP participation	Key architectural innovation	Primary prediction scope	Benchmark achievement
AlphaFold 1 (AF1)	CASP13 (2018)	DNN, Co-evolutionary couplings, contact maps	Single protein chains (initial fold modeling)	Ranked first in free modeling (FM)
AlphaFold 2 (AF2)	CASP14 (2020)	End-to-end DL, evoformer, high-resolution refinement	Single protein chains, multimers	Highest score achieved (92.4 GDT)
AlphaFold 3 (AF3)	Released 2024	Pairformer, unified model of life’s molecules	Proteins, DNA, RNA, ligands, ions, modified residues	50% more accurate than best traditional methods (PoseBusters)

## 3 Comparison between AlphaFold 1 (AF1), AlphaFold 2 (AF2), and AlphaFold 3 (AF3)

AlphaFold has evolved into three versions: AF1, AF2, and AF3. To trace their progression, AF1 was demonstrated at CASP13 in 2018, AF2 at CASP14 in 2020, and AF3 in 2024. Starting with AF1, deep learning was used to predict protein structures with moderate accuracy. In 2018, AF1 introduced a deep learning approach using convolutional neural networks to predict inter-residue distances and torsion angles. Although it achieved high accuracy in the CASP13 competition, it remained limited (AlQuraishi, 2019; Peng et al., 2025).

Building on advances in AF1, AF2 achieved greater accuracy and solved the protein folding problem for single proteins. In 2020, it revolutionized the field with its transformer-based architecture and a median GDT score above 90. This breakthrough earned recognition in structural biology (Jumper et al., 2021a). AF3 further expanded the technology to predict complex interactions among proteins, DNA, RNA, and small molecules. In 2024, AF3 was extended to predict the structures of biomolecular assemblies, including protein–DNA, protein–RNA, protein–ligand interactions, and post-translational modifications. AF3 also uses a diffusion-based model, directly generating atomic coordinates to determine exact atom positions (Abramson et al., 2024).

In summary, each AlphaFold version is built on its predecessor. AF1 used deep neural networks. AF2 employed the Evoformer, designed to model related protein sequences. AF3 used the Pairformer, a model developed to capture pairwise interactions between amino acid residues. All AlphaFold versions applied deep neural networks to generate structures from amino acid sequences; their main difference was architecture. AF1 showed deep learning could predict protein structures. AF2 improved on AF1’s accuracy with the same approach. AF3 further extended these capabilities to predict interactions between different molecules (Table 2).

## 4 The post-AlphaFold landscape and global impact

AlphaFold, particularly AF2, has rapidly transformed the field of structural biology. During the era of AlphaFold (AF1/AF2/AF3), several AlphaFold-associated tools have been developed and applied in various areas of biological science, particularly in structural biology, by researchers across different fields (Table 3). It has significantly influenced the way we associate sequences with structures.

**Table 3:** AlphaFold and different AlphaFold-associated tools and their different applications used by researchers for structural biology from time to time.

Sl. No.	Name of the tool	Category/type	Remarks	Reference
1.	ModelArchive	Database	The public repository for depositing 3D protein structure models	Tauriello et al. (2025)
2.	AlphaBridge	Evaluation and visualization	It designed to analyze and post-process predicted macromolecular complex models to deduce and visualize interaction interfaces	Álvarez-Salmoral et al.(2024)
3.	SubtiWiki	Structure comparison	The curated, community-oriented wiki/database focused on the *Bacillus subtilis* model organism	Elfmann et al. (2025)
4.	ColabFold	Structure prediction	The accessible interface (Google Colab notebook + command-line tool) for running AlphaFold2 (and related models) more easily and efficiently	Mirdita et al. (2022)
5.	AlphaFold clusters	Structure comparison	The sets of protein structures from the AlphaFold Protein Structure Database that share similar folds or overall 3D shapes	Barrio-Hernandez et al. (2023)
6.	PAE viewer	Visualization and evaluation	The interactive visualization tool used to explore the confidence and relative accuracy of AlphaFold protein structure predictions	Elfmann and Stulke (2023)
7.	AlphaFold server	Structure prediction	The public web platform that allows researchers to generate protein structure predictions using DeepMind’s AlphaFold2 system	Abramson et al. (2024)
8.	Foldseek search	Structure comparison	The fast, structure-based search tool that allows to compare 3D protein structures — similar to how BLAST compares sequence	van Kempen et al. (2024)
9.	AlphaFold DB	Database	The public database that provides AI-predicted 3D structures of proteins for nearly all known sequences in nature	Varadi et al. (2024)

### 4.1 Structural coverage expansion

AlphaFold has changed the structural landscape of the human proteome, increasing structural coverage. Research demonstrates that the structural coverage of all human protein residues increased from 48% before AlphaFold’s implementation to 76% thereafter (Porta-Pardo et al., 2022). Structural coverage is the key finding of this research work. Here, using three-dimensional coordinate files from the PDB, the coverage of the human proteome was evaluated. In this context, structural coverage refers to the percentage of the human proteome for which we have known or predicted three-dimensional protein structures. More specifically, it measures the proportion of all human proteins (or protein residues) that have available structural information, estimated either from experimental structures determined by techniques such as X-ray crystallography or cryo-EM, or from computational models (homology modelling) or AI predictions (from AF). The study’s key finding is that structural coverage increased from 48 to 76% when AF predictions were included. While the “dark proteome” (proteins without any structural information) decreased from 26% to just 10%. At the protein level, the number of human proteins completely lacking structural coverage dropped from 5,027 to just 29 proteins after AF’s release. Essentially, structural coverage is a measure of how much of the proteome we can visualize and understand at the three-dimensional level. It is critical for understanding protein function, disease mechanisms, and drug design. This augmentation represents a significant advancement in fundamental biological knowledge. The number of high-quality models has significantly increased. Models exhibiting a sequence identity of 50% or greater with a Protein Data Bank (PDB) chain have risen from 31 to 50%. AlphaFold has nearly eliminated the “dark proteome.” The “dark proteome” refers to the collection of protein regions and entire proteins whose three-dimensional (3D) structures are largely unknown and cannot be determined experimentally or computationally, such as through homology modeling.

In structural biology, the number of unsolved human proteins has dropped dramatically, representing a revolution in the field. Obtaining, interpreting, and validating a structure once took years, but now researchers can focus more on protein function, interactions, and dynamics (Alderson et al., 2023).

### 4.2 Data democratization and the AlphaFold database (AFDB)

Data democratization is the process of making data accessible to everyone, so it can be utilized effectively and efficiently. This approach empowers individuals to make informed decisions (Lefebvre et al., 2021; Marcu et al., 2022; Mseer and Al Makhzoumi, 2025), highlighting an urgent need for data and knowledge democratization (Dessimoz and Thomas, 2024). AlphaFold exemplifies this by making structural data more accessible, with data availability as a core component. The AlphaFold Protein Structure Database (AFDB) began in 2021 with 300,000 structures and has grown to over 214 million predicted protein structures (Varadi et al., 2022; Varadi et al., 2024), making it 500 times larger and comprising a significant portion of the UniProt repository. AFDB is an open-access online repository that provides detailed protein structures to scientists worldwide, fostering a sustainable and innovative research landscape. For instance, Nji et al. (2025) indicate it has a positive impact on African healthcare research, supporting researchers in evaluating model accuracy.

Additionally, AFDB offers atomic-level details that greatly benefit drug development and disease research (Varadi and Velankar, 2023; Chang et al., 2024; Desai et al., 2024). Used by over 2 million individuals in 190 countries, the library saves researchers significant time and financial resources. This diverse information accelerates scientific discoveries, especially given the dramatic increase in data availability observed from the pre- to post-AlphaFold eras, as shown in Table 4.

**Table 4:** Quantitative impact on human structural coverage during the pre-AlphaFold era and the post-AlphaFold era.

Metric	Pre-AlphaFold (Approximate)	Post-AlphaFold (AFDB 2024)	Significance
Human proteome coverage (All residues)	48%	76%	Major expansion of structural knowledge base.
Proteins with no structural coverage	5,027	29	Near elimination of “dark” disease targets.
AFDB structure count	300 k (2021 initial release)	>214 million	500-fold expansion, enabling global access.
High-quality coverage (≥50% Seq. ID)	31%	50%	Increase in clinically relevant, accurate models.
Reference	Porta-Pardo et al. (2022), Varadi and Velankar (2023), and Varadi et al. (2024)	Porta-Pardo et al. (2022), Varadi and Velankar (2023), and Varadi et al. (2024)	-

### 4.3 The association between the AlphaFold, AFDB, and UniProt

While calling AlphaFold “democratizing access to structural data” captures its broad impact, it also obscures the specific methodological advances behind this shift. The AlphaFold Protein Structure Database (AFDB) provides open access to hundreds of millions of high-accuracy predicted protein structures (Jumper et al., 2021b). It is now more closely aligned with UniProt sequence releases. This change embeds structural models within the primary sequence knowledgebase used across biology and improves integration with common data resources (Varadi et al., 2024). AFDB predictions are now cross-referenced with UniProt and related resources, improving their usability for functional annotation and bioinformatics workflows. The open-source release of the AlphaFold code also allows users to run predictions on novel custom sequences beyond those already in AFDB. This has greatly expanded practical access to structure prediction (Lang et al., 2025).

### 4.4 The 2024 Nobel prize in chemistry was given to AlphaFold

AlphaFold’s scientific contribution was acknowledged when its developers, Demis Hassabis and John Jumper of Google’s DeepMind, were awarded half of the Nobel Prize (Chemistry) for their work in “predicting protein structures.” The award, shared with David Baker for his work in “designing proteins using computers” (Callaway, 2024; Graham, 2024; Najar Najafi et al., 2025), highlights AlphaFold’s capabilities and innovative protein design approach. AlphaFold’s accurate predictions help researchers design novel proteins efficiently and demonstrate the importance of integrating extensive public data resources, such as the PDB, with new AI developments. This recognition may trigger the evolution of biology with AI models (Abriata, 2024).

In addition, David Baker was recognized for his achievements in protein design, a computational method that is part of AlphaFold. He developed a competing approach, called RoseTTAFold, for computational protein design (Mansoor et al., 2023).

## 5 Alphafold’s translational research applications

**Figure 2:**
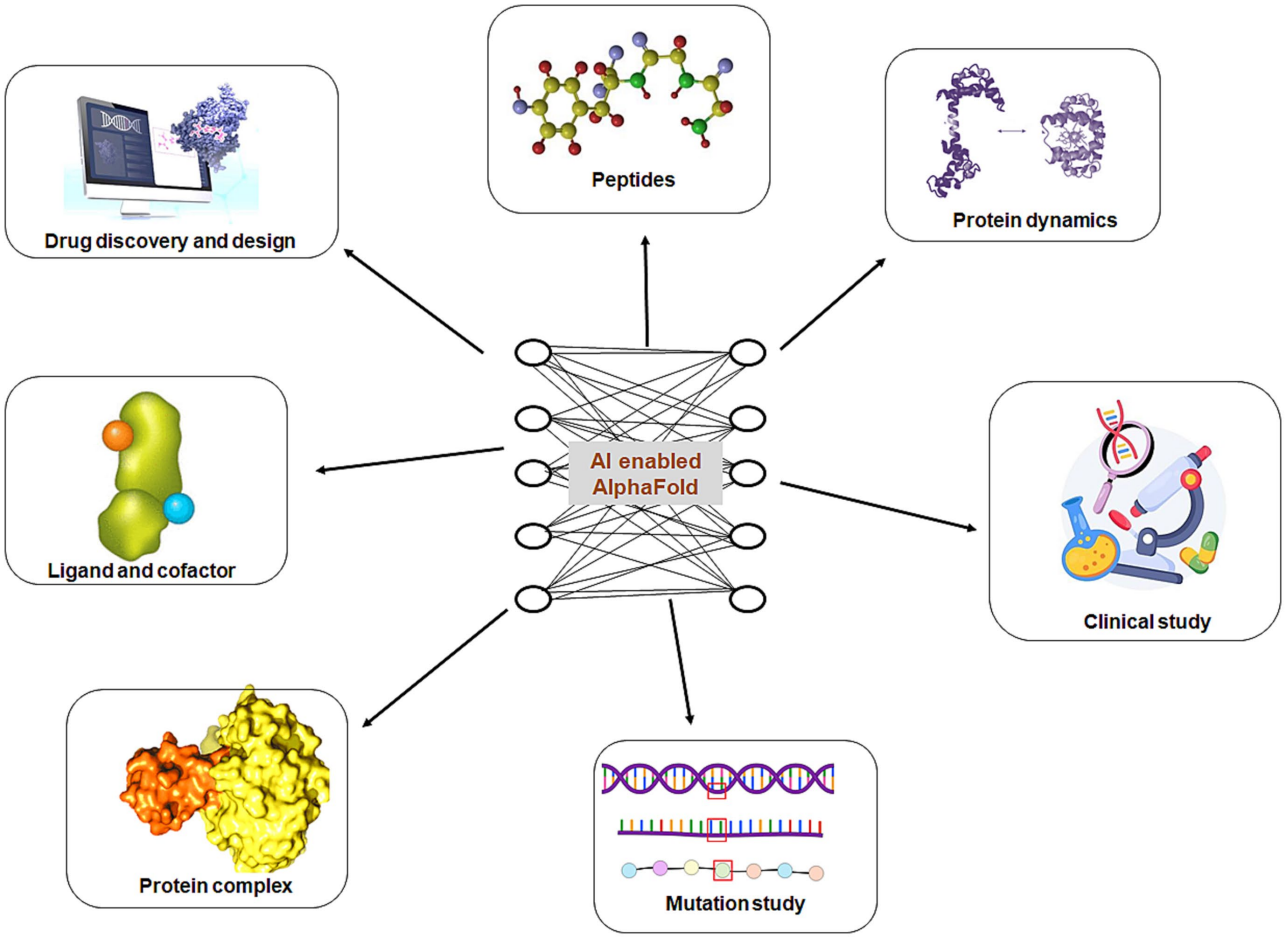
AlphaFold applications in different translational research in the field of biological and medical discovery.

AlphaFold serves as an essential platform that facilitates biological and medical discoveries across a wide range of applied fields (Figure 2).

### 5.1 AlphaFold helps in the advancements in structure-based drug discovery (SBDD)

The detailed shape of a protein is key to designing drugs. AlphaFold has quickly given high-quality structure predictions for many hard-to-study targets. Now, these targets can be used for structure-based drug research (Jumper et al., 2021b; Nussinov et al., 2023; Guo et al., 2024).

Recently, researchers found that AlphaFold achieved a 60% hit rate when predicting protein structures for TAAR1 agonists, outperforming conventional methods. In this context, a hit was defined as a tested compound that showed agonist activity. AlphaFold’s virtual screen identified 30 top-ranked compounds, of which 18 displayed agonist activity, with potencies ranging from 12 to 0.03 μM. In contrast, traditional homology modeling identified 32 top compounds, but only 7 were active, yielding a 22% hit rate. These results indicate AlphaFold’s clear advantage in hit identification. However, while AF2 models predict binding pockets with high accuracy, their ability to predict ligand-binding poses via computational docking is comparable to that of traditional methods and lower than that of docking to experimentally determined structures. This poses challenges for structure–activity relationship (SAR) studies and lead optimization, as accurately predicting subtle structural modifications remains difficult. Additionally, AlphaFold models are limited by their lack of structural plasticity, typically offering only one conformational state, which may not reflect ligand-bound forms. Therefore, further structure refinement is needed. Despite these limitations, AlphaFold remains a valuable tool because it generates predictions even for proteins with low sequence identity to known templates, particularly for those with less than 20% sequence similarity.

AlphaFold3 enriches the drug design landscape by predicting interactions among ligands, ions, and antibodies. It is used to assess variations in binding energy and evaluate the impact of mutations on protein interactions, which are helpful in developing novel therapeutic approaches such as targeted protein degradation (He et al., 2025; Szczepski and Jaremko, 2025; Zhong et al., 2022; Mathur et al., 2025; Xue et al., 2025).

AF3 can predict interactions among proteins, ligands, antibodies, and other biomolecules for drug design. In benchmark datasets, AF3 reached 76.4% accuracy in protein-ligand docking—a 1.8-fold improvement over prior approaches. It also showed 50% greater accuracy than physics-based methods. However, key limitations remain. Evaluations show AF3 struggles to predict ligand-binding poses for ions and peptides in targets such as GPCRs, and it displays minimal correlation between predicted and experimental binding affinities. AF3 also performs poorly on test sets post-training, suggesting memorization rather than genuine modeling. For targeted protein degradation, such as in PROTACs, AF3 can predict ternary complex structures if given explicit ligand details, with recent studies showing 33 complexes predicted to sub-Ångström accuracy. Yet it still struggles to accurately model small protein–protein interfaces typical of such interactions. These findings show AF3’s strength in developing structural hypotheses and visualizing validated binding pairs, while also emphasizing key areas needing improvement.

### 5.2 Resolving large, challenging macromolecular assemblies

AF2-Multimer and AF3 have advanced the study of large, complex assemblies (Ibrahim et al., 2023; Yang et al., 2023; Abramson et al., 2024), which have long challenged experimental techniques due to their size, flexibility, or diversity.

Building on these advances, the use of AlphaFold has brought a significant change to the structural study of the human nuclear pore complex (NPC). This substantial structure is crucial for the nucleus in eukaryotic cells and comprises approximately 1,000 proteins of around 30 distinct types. With AlphaFold structures, researchers have explained approximately 90% of the human NPC structure, providing insights into cellular machinery (Lin and Hoelz, 2019; Fontana et al., 2022; Mosalaganti et al., 2022; Fleming et al., 2025). Furthermore, AlphaFold has facilitated the determination of the Mce1 structure, a pivotal protein used by the tuberculosis bacterium to acquire nutrients from host cells, increasing the understanding of disease mechanisms (Chen et al., 2023; Kovalevskiy et al., 2024; Rennie and Oliver, 2025). Its rapid modeling capability accelerates research in critical health domains and is now referred to as “science at digital speed” (Elfmann and Stulke, 2025; Rennie and Oliver, 2025). Additionally, AlphaFold has enabled researchers to advance investigations beyond static structures by modeling dynamic protein conformations (Cui et al., 2025). Ultimately, AlphaFold represents a transformative leap in structural biology, driving new discoveries and redefining our approach to understanding complex biological assemblies.

### 5.3 Protein engineering and synthetic biology

In the domain of protein engineering, AlphaFold provides high-precision and rapid structural validation. Therefore, it significantly reduced the design-build-test cycle duration (Kovalevskiy et al., 2024; Matzko and Konur, 2024; Uzoeto et al., 2024). By generating testable hypotheses about how sequence modifications translate into altered three-dimensional conformations, researchers can rationally design novel protein functions. Applications encompass the development of more effective pharmaceuticals and the creation of novel enzymes capable of degrading plastic pollution. AF3 has specific capabilities, such as predicting physiologically relevant metal-binding sites and modelling complex β-solenoid structures. Furthermore, it enhances the capacity to engineer novel proteins with precise functional requirements (Marcu et al., 2022; Krokidis et al., 2025).

Collectively, these advances signal a foundational shift: instead of relying solely on empirical trial-and-error, researchers now leverage predictive, computationally guided strategies for protein and enzyme design. This integrated approach—with generative AI, structural insights, and experimental validation—unlocks the creation of programmable biocatalysts and broadens possibilities in medicine, energy, and environmental applications, marking a new era in synthetic biology.

### 5.4 AlphaFold-predicted structures and their other downstream applications

Presently, AlphaFold (AF) structures are used in many downstream assessments and applications, including protein function, druggability, stability, protein interfaces, and evolutionary relationships. Researchers use AF structures in different orientations to map protein function. This includes identifying active sites, binding pockets, and functional domains (Castillo and Ollila, 2025). In drug discovery, AF models help assess druggability and enable virtual screening for candidates (Alhumaid and Tawfik, 2024). Predicted structures are also useful for evaluating protein stability and detecting destabilizing mutations (Pak et al., 2023). AF structures provide insight into protein–protein interaction networks and their interfaces (Lee et al., 2024). They also help researchers understand signaling pathways and complex formations (Li et al., 2025a). In addition, AF predictions reveal evolutionary relationships among proteins (Jumper et al., 2021b). Comparative analyses show conserved structural motifs and functional adaptations in homologous proteins (Ruperti et al., 2023).

### 5.5 Several AlphaFold variants and alternatives in protein structure prediction

Several AlphaFold variants and alternative methods have emerged for protein structure prediction, each serving distinct use cases. ColabFold is a free platform for rapid structure prediction that employs AlphaFold2 and MMseqs2 to accelerate multiple sequence alignment and structure modeling, accessible via Google Colaboratory or the command line. It offers predictions about 5 times faster than standard AlphaFold2 (Kim, Gyuri et al., 2025; Mirdita et al., 2022). In contrast, the AlphaFold Server provides free, non-commercial access to AlphaFold 3, with a limit of 30 jobs per day, to promote accessibility. AlphaFold-Multimer (AFM) extends AlphaFold2 by enabling the prediction of protein complexes and multi-chain assemblies (Zhu et al., 2023; Homma et al., 2023). OmegaFold differs by predicting high-resolution protein structures from a single sequence through protein language models, without requiring evolutionary information (Jing et al., 2024; Hýskova et al., 2025; Wu et al., 2022). RoseTTAFold uses a neural network to achieve high-accuracy structure prediction comparable to AlphaFold2 and provides an alternative method for researchers (Krishna et al., 2024; Lisanza et al., 2025; Liang et al., 2022; Barbarin-Bocahu and Graille, 2022).

## 6 Limitations and next-generation challenges

AlphaFold has addressed the problem of protein folding. However, it continues to pose obstacles in the field of structural biology research. These challenges arise from the PDB training data, which primarily consists of standard structures. Consequently, while AlphaFold excels at analyzing known structures, it struggles with novel or dynamic structures.

### 6.1 The challenge of protein dynamics and multiple conformations

A significant limitation of AF2, and to a lesser extent AF3, is their tendency to predict a singular, thermodynamically stable ground state (Laurents, 2022; Chakravarty et al., 2024). This makes it difficult to assess protein structures with different conformational states. Many proteins exist in multiple conformational states (Cui et al., 2025). Proteins often change conformation during ligand binding, catalysis, or interactions with biomolecules (Plattner and Noé, 2015). These changes are critical for biological function (Liu et al., 2008). However, AF2 or AF3 predictions cannot capture these dynamic states (Perkins-Jechow et al., 2025). This fixed state is challenging to understand. Various biological processes, such as enzyme catalysis, signal transduction, and membrane transport, rely on fold-switching or substantial conformational changes. These processes involve transitions among distinct stable, metastable, and transient states (Bryan and Orban, 2010; Chakravarty et al., 2023). For example, AlphaFold accurately predicts only the closed state of Adenylate kinase. It thereby neglects the open state, which is functionally critical and has been experimentally observed (Guan et al., 2024; Lee et al., 2025b). Moreover, AlphaFold faces several challenges when predicting fine-grained functional effects, such as those caused by point mutations. It cannot model transient or excited states. Researchers can assess model quality, but high-confidence metrics (pLDDT > 90) suggest only a high probability of accurate local coordinate positioning (pLDDT, 2024). This does not guarantee concordance with the native protein conformation if that conformation is dynamic, involves multiple states, or belongs to a non-globular class. Confidence metrics serve as estimators of coordinate likelihood. They are not predictors of native functional relevance for flexible systems (Sala et al., 2023).

### 6.2 Noncanonical chemistry and unseen residues

AlphaFold, when it predicts protein structures based on evolutionary principles, encounters significant challenges. This is especially true for proteins that incorporate Noncanonical Amino Acids (NCAs) or exhibit complex Post-Translational Modifications (PTMs) (Yang et al., 2023; Agarwal and McShan, 2024). It often fails to distinguish NCAAs from the input data and MSAs. Here, generic “X” tokens were represented in sequence databases. In biological sequence databases, the generic “X” token typically represents an unknown or undetermined residue (Kemena and Notredame, 2009; Castro et al., 2023; Agarwal and McShan, 2024; Dotan et al., 2024; Testagrose and Boucher, 2025). This representation obscures their identity and hinders the model’s ability to extract relevant co-evolutionary signals.

Challenges with evolutionary-based models arise when achieving optimal performance for complex and novel chemical structures.

Specifically, for peptides such as sactipeptides, which are characterized by unique sulfur-to-alpha-carbon linkages, these models yield low predictive scores ranging from 0.0 to 19.2%. This suggests their inability to accurately predict structures in some cases (Fluhe and Marahiel, 2013; Chen et al., 2021; Agarwal and McShan, 2024). Consequently, it highlights the need for models that incorporate physical principles to address the complexities of chemical systems, surpassing the capabilities of evolution-based models (Wee and Wei, 2024a).

### 6.3 Accuracy in complex assemblies and stereochemistry

AF3 can develop complex molecular structures, but the resulting structures sometimes contain errors. For example, compared to original Protein Data Bank (PDB) complexes, AF3 predictions show an 8.6% error in binding free energy changes (ΔΔG) (Wee and Wei, 2024b). AF3 can also cause overlapping atoms, especially in proteins with multiple identical chains, leading to inaccuracies. Additionally, the server struggles to maintain the correct three-dimensional (3D) conformation of small molecules in drug research (Schreiner et al., 2011; Steinkellner et al., 2025). According to the PoseBusters test, chirality errors occur at a rate of 4.4%. Thus, even with precise chemical input, AF3’s generalized approach may not match the accuracy of specialized methods.

For complex assemblies, AF3 shows moderate performance, with success rates ranging from 40 to 60% across different types of complexity (AlphaFold, 2025). Accuracy decreases for larger heteromeric assemblies. The model achieved approximately 76% accuracy in predicting protein complexes that involve intrinsically disordered regions (Gopalan and Narayanan, 2025). However, performance drops significantly when evaluated using interface-specific metrics, such as DockQ (which measures the quality of predicted protein–protein interfaces), compared to global structural metrics (which assess the overall similarity between predicted and experimental structures). For stereochemistry, AF3 exhibits a 4.4% chirality violation rate on the PoseBusters benchmark (Gopalan and Narayanan, 2025; Childs et al., 2025; Abramson et al., 2024). This occurs despite the incorporation of penalty mechanisms for chiral errors. The model also produces significant errors in ligand bond lengths and bond angles (Ishitani and Moriwaki, 2025). Systematic issues include incorrect chirality assignments even when provided with correct reference structures. These stereochemical errors particularly affect small-molecule predictions, which are critical for drug design applications (Ishitani and Moriwaki, 2025).

### 6.4 Intrinsically disordered proteins

AlphaFold also does not handle several issues appropriately, including the structures of intrinsically disordered proteins (IDPs). In higher organisms, IDPs account for approximately one-third of proteins (Pancsa and Tompa, 2012). Because IDPs lack stable three-dimensional structures and exist as dynamic conformational changes, they cannot be modeled by AF (Ruff and Pappu, 2021). This incompatibility stems from AF’s focus on stable structures. Nevertheless, AF’s inability to reliably predict a single structure may itself indicate intrinsic disorder, effectively making AF an excellent predictor of structural disorder (Akdel et al., 2022). As a result, this characteristic might prove significant for AF.

### 6.5 Data leakage

AlphaFold performs very well at structure prediction. Because of this, many researchers use AlphaFold for applications beyond simple structure prediction. However, they often neglect a significant statistical error. This error occurs when the same structures are included in both training and analysis (Dobson et al., 2025).

Data leakage occurs when there is overlap between AlphaFold’s training and test datasets. More specifically, in AF, data leakage typically occurs when the training data for PDB overlaps with the validation or test sets. As a result, it causes inflated performance metrics due to high sequence similarity (Verburgt et al., 2025; Bernett et al., 2024). When test proteins are structurally similar to those in the training set, this overlap can further exaggerate performance. Additionally, AF3’s test set uses time-based splits without ligand similarity filtering, which makes it difficult to assess performance on truly novel ligands and binding pockets. Consequently, this raises concerns about whether AlphaFold relies on memorization of training data rather than understanding protein-ligand chemistry (Masters et al., 2025; Outeiral et al., 2022; Škrinjar et al., 2025).

### 6.6 An overview of the technical limitations of AlphaFold (AF1/AF2/AF3) affects biological interpretation and translational workflows, addressing challenges related to intrinsically disordered regions, post-translational modifications, and transient interactions

There are limitations to AlphaFold (AF1/AF2/AF3) that significantly affect biological interpretation and translational workflows, so users must carefully consider their appropriate use. Notably, all AlphaFold versions encounter difficulties with intrinsically disordered regions (IDRs), which comprise 30–40% of the human proteome (Gopalan and Narayanan, 2025) and play critical roles in cellular signaling and disease. For example, pLDDT confidence scores of AF2 correlate with intrinsic disorder (Alderson et al., 2023), enabling the server to identify IDRs; however, these models cannot evaluate the dynamic nature and conformational heterogeneity of these regions, and, in some cases, AF3 incorrectly predicts ordered structures for 22% of disordered residues (Gopalan and Narayanan, 2025). Furthermore, post-translational modifications (PTMs) represent another critical limitation relevant to IDRs.

Challenges extend to PTMs as well: AF2 was not designed to model PTMs and cannot predict their structural impact. Recent evidence suggests PTMs may be the key factor influencing IDR folding (Bah and Forman-Kay, 2016). AlphaFold also struggles with protein–protein interactions; its training may favor high-affinity, homogeneous binding, potentially hindering predictions of cellular assembly. Furthermore, user education on confidence metrics is essential. For example, a high pLDDT score does not always reflect structural accuracy. Membrane proteins may receive very high confidence scores, yet such predictions can deviate from experimental structures. Systematic data bias further complicates structure determination and downstream applications. PDB training data, for instance, skews toward soluble proteins—less than 3% are membrane proteins (Ahram et al., 2006)—and toward well-studied families and static conformations. AlphaFold predictions tend to regularize regions of conformation, yet conformational diversity may differ greatly from experimentally determined structures. Thus, AlphaFold predictions should supplement, not supplant, experimental validation. This is especially important for drug discovery, protein engineering, and studies of disease mechanisms, where structural dynamics, PTMs, and transient interactions are critical.

## 7 The future trajectory: integrating AI with modelling

AlphaFold exhibits certain limitations, reflecting the current state of structural biology. Presently, scientists employ a combination of methodologies that integrate rapid and precise deep learning techniques with realistic physical models to investigate biological processes.

### 7.1 Predictions of single-chain structures

It has been claimed that single-chain structure prediction is mostly solved by AF3. However, this needs qualification. While AF3 shows better local structural accuracy than AlphaFold2, its global accuracy improvements for protein monomers are limited. For example, AF3 outperformed AF2 in only 57.5% of cases on orphan protein benchmarks (Peng et al., 2025). The most challenging cases are orphan proteins without detectable multiple sequence alignments. In these instances, AF2 actually performs better than AF3 in both global and local predictions. Thus, AF3’s accuracy has not increased significantly for protein monomer structure prediction compared to AF2 (Peng et al., 2025), especially for orphan proteins lacking sequence homology. As a result, predictions for these proteins are often unsatisfactory (Singh, 2023). In summary, the persistent shortcomings of current models, particularly regarding orphan proteins, underscore that single-chain structure prediction is far from solved and remains a critical challenge that demands further advances.

### 7.2 Methods for conformational sampling and ensemble generation

Recent research shows that protein sequences alone carry the information for conformational changes. This can be seen using MSA (multiple sequence alignment), a method for comparing protein sequences. Researchers have developed new techniques—MSA masking (hiding parts of the alignment to test their importance), subsampling (analyzing random subsets), and clustering (grouping similar sequences)—to generate diverse sub-MSAs with minor changes (Genc and McGuffin, 2025; Kalakoti and Wallner, 2025). These help AlphaFold generate a range of possible protein shapes, not just a single static structure. Also, new models use diffusion (iterative denoising) and flow-matching (aligning distributions of structures) techniques. These methods are transforming protein structure prediction (Sala et al., 2023). As a result, models can now predict multiple molecular shapes, helping us understand more than a single state (Zheng et al., 2023; Genc and McGuffin, 2025; Kalakoti and Wallner, 2025; Li et al., 2025b).

### 7.3 Post-prediction refinement using molecular dynamics (MD) simulations

AI and physics-based computational methods are crucial for studying molecular energy states. AlphaFold efficiently identifies the lowest-energy state of molecular structures (Perrakis and Sixma, 2021; Zhang et al., 2024). In contrast, Molecular Dynamics (MD) simulations provide essential insights into the energy landscape and molecular conformations. Studies show that combining AlphaFold with MD simulations improves accuracy, demonstrating that this synergy outperforms AI alone (Heo and Feig, 2020; Elia Venanzi et al., 2024). This approach is particularly useful for modeling conformational changes affecting molecular function. By starting MD simulations with AlphaFold predictions, researchers can explore alternative conformations; specific transporter structures have been examined using this strategy (Heo and Feig, 2020; Elia Venanzi et al., 2024; Guo et al., 2024). These methods also help model transient states, intermediates between two functional conformations. Thus, integrating AI predictions with MD simulations greatly enhances our understanding of protein conformational changes.

### 7.4 Advanced benchmarking for multi-molecular systems

There is a need for more refined methods with thoroughly evaluated performance. In complex prediction, innovative models—such as combining AF3 with robust predictors like Protenix—have achieved 70–80% success rates under strict conditions (Fnat ≥ 0.8), much higher than AlphaFold2-multimer’s 53% (Varga et al., 2025; Zhou et al., 2025). Researchers are also working to improve model quality assessment beyond traditional metrics like iPTM and RMSD. Evaluating concordance among models has become an effective alternative (Varga et al., 2025). Defining these measures is crucial, especially for flexible or complex targets, since metrics like iPTM may not capture structural misalignments.

Building on recent advances and ongoing challenges, we outline current limitations and mitigation or hybrid strategies to enhance dynamic modelling and multi-molecular systems, as shown in Table 5. These insights highlight pivotal directions for overcoming limitations and advancing the field.

**Table 5:** Current limitations and strategies for dynamic modelling and multi-molecular systems.

Challenge area	Specific limitation/artifact	AlphaFold context	Mitigation/hybrid strategy
Protein dynamics	Multiple conformations, transient states, fold-switching	AF2 and AF3 are biased toward a static ground state	MD simulations, MSA subsampling/enhanced sampling, generative models
Non-canonical chemistry	NCAAs, complex PTMs (e.g., sactipeptides)	Evolution-based models fail where MSA signals are masked or absent	Physics-informed models, explicit chemical tokenization (e.g., RareFold)
Small molecule accuracy	Stereochemistry/Chirality Violations	AF3 exhibits 4.4% chirality violation rate	Required physics-based refinement or customized chemical modules
Model quality assessment	High confidence (pLDDT) deviation from non-globular native structure	Confidence metrics do not guarantee functional relevance for highly flexible regions	Updated error categorization; focus on PAE for relative domain orientation

## 8 Conclusion

AlphaFold has fundamentally revolutionized structural biology, transforming how scientists predict and decipher protein structures with unprecedented accuracy. In recognition of this extraordinary breakthrough, the Nobel Prize was awarded to Demis Hassabis and John Jumper (Callaway, 2024; Graham, 2024). AlphaFold has democratized access to structural data, expanding the AlphaFold Database (AFDB) to an astonishing 214 million+ entries (Varadi et al., 2024). Moreover, structural coverage of the human proteome increased from 48 to 76% (Porta-Pardo et al., 2022; Varadi and Velankar, 2023).

Building on AlphaFold’s transformative impact, advancements in this domain have shifted the field’s focus. The prediction of single-chain structures is largely resolved. The current significant challenge lies in modelling biological functions under real-life conditions.

To address these ongoing challenges, it is crucial to apply AI models by investigating various properties of proteins, such as dynamics, conformational changes, and molecular interactions. Accordingly, the focus of future research is on integrating DL, which excels at rapid identification of protein conformations, with physics-based methodologies such as Molecular Dynamics. These approaches can explain various aspects of protein behavior using distinct parameters. The strategy is expected to yield significant advances in protein structure and is of great importance to both the field of biotechnology and medicine.
